# Dendrogram of transparent feature importance machine learning statistics to classify associations for heart failure: A reanalysis of a retrospective cohort study of the Medical Information Mart for Intensive Care III (MIMIC-III) database

**DOI:** 10.1371/journal.pone.0288819

**Published:** 2023-07-20

**Authors:** Alexander A. Huang, Samuel Y. Huang

**Affiliations:** 1 Department of MD Education, Northwestern University Feinberg School of Medicine, Chicago, IL, United States of America; 2 Department of Internal Medicine, Virginia Commonwealth University School of Medicine, Richmond, VA, United States of America; Jeonbuk National University, REPUBLIC OF KOREA

## Abstract

**Background:**

There is a continual push for developing accurate predictors for Intensive Care Unit (ICU) admitted heart failure (HF) patients and in-hospital mortality.

**Objective:**

The study aimed to utilize transparent machine learning and create hierarchical clustering of key predictors based off of model importance statistics gain, cover, and frequency.

**Methods:**

Inclusion criteria of complete patient information for in-hospital mortality in the ICU with HF from the MIMIC-III database were randomly divided into a training (n = 941, 80%) and test (n = 235, 20%). A grid search was set to find hyperparameters. Machine Learning with XGBoost were used to predict mortality followed by feature importance with Shapely Additive Explanations (SHAP) and hierarchical clustering of model metrics with a dendrogram and heat map.

**Results:**

Of the 1,176 heart failure ICU patients that met inclusion criteria for the study, 558 (47.5%) were males. The mean age was 74.05 (SD = 12.85). XGBoost model had an area under the receiver operator curve of 0.662. The highest overall SHAP explanations were urine output, leukocytes, bicarbonate, and platelets. Average urine output was 1899.28 (SD = 1272.36) mL/day with the hospital mortality group having 1345.97 (SD = 1136.58) mL/day and the group without hospital mortality having 1986.91 (SD = 1271.16) mL/day. The average leukocyte count in the cohort was 10.72 (SD = 5.23) cells per microliter. For the hospital mortality group the leukocyte count was 13.47 (SD = 7.42) cells per microliter and for the group without hospital mortality the leukocyte count was 10.28 (SD = 4.66) cells per microliter. The average bicarbonate value was 26.91 (SD = 5.17) mEq/L. Amongst the group with hospital mortality the average bicarbonate value was 24.00 (SD = 5.42) mEq/L. Amongst the group without hospital mortality the average bicarbonate value was 27.37 (SD = 4.98) mEq/L. The average platelet value was 241.52 platelets per microliter. For the group with hospital mortality the average platelet value was 216.21 platelets per microliter. For the group without hospital mortality the average platelet value was 245.47 platelets per microliter. Cluster 1 of the dendrogram grouped the temperature, platelets, urine output, Saturation of partial pressure of Oxygen (SPO2), Leukocyte count, lymphocyte count, bicarbonate, anion gap, respiratory rate, PCO2, BMI, and age as most similar in having the highest aggregate gain, cover, and frequency metrics.

**Conclusion:**

Machine Learning models that incorporate dendrograms and heat maps can offer additional summaries of model statistics in differentiating factors between in patient ICU mortality in heart failure patients.

## Introduction

Heart failure is a condition that affects a growing number of people and is one of the leading causes of death and hospitalization [1,2]. Patients with heart failure may need to spend more time in the hospital because there aren’t many options for managing their condition [3–5]. This is especially true for those with acute heart failure in the intensive care unit (ICU), where multiple underlying conditions may make their stay longer [6,7]. The financial burden of acute heart failure can have a significant impact on patient quality of life and is significant [8,9]. For many heart failure patients, particularly those with advanced organ dysfunction or severe complications, ICUs are necessary to provide advanced, high-tech, life-saving care [10,11]. ICUs have a high-intensity staffing model with high nurse and physician-to-patient ratios [12,13]. In the USA, approximately 10%–51% of hospitalized heart failure patients are admitted to an ICU [14,15]. ICU-admitted patients have significantly higher adjusted in-hospital mortality rates compared to those admitted to hospital wards only [16,17]. The in-hospital mortality rate for ICU-treated patients has been reported as 10.6%, whereas the rate for all HF patients is 4.0% [18,19]. Therefore, accurately predicting prognosis and providing intensive treatment with closer follow-up may be of greater benefit to ICU-admitted heart failure patients [20,21]. Although several in-hospital mortality prediction models are available, they lack model transparency and feature importance. Moreover, limited data are available on prediction models for ICU-admitted heart failure patients.

The use of machine learning in medicine for developing highly precise predictive models is on the rise [22–24]. To achieve this, a common approach involves utilizing the XGBoost algorithm, which is known for its high accuracy, along with the transparent Shapely Additive Explanations (SHAP) algorithm to determine crucial covariates and their predictive direction [25,26]. In our research, we expanded upon this approach by integrating dendrograms and heatmaps to visually summarize covariates based on their gain, cover, and frequency. In the context of explainable machine learning, dendrograms provide additional insights by showing the relationships between variables based on their similarity, allowing for easier identification of important factors and potential interactions.

Table 1 compares the use of both SHAP and dendrograms, but ultimately how the use of both can provide additional information. SHAP values help us understand the impact of each feature on predictions, while dendrograms enhance model transparency by visualizing patterns and relationships among variables. Their application can support feature selection, model understanding, and decision-making processes in various domains. Eqs 1 and 2 similarly makes the above point using mathematical formulas. Eq 1 shows the general formula for calculating SHAP value as follows:

$$g(x^{\prime })=\Phi_{0}+\sum_{j=1}^{M}\Phi_{j}z_{j}^{\prime }$$ (1)

**Table 1:** Comparison of the application of SHAP values and dendrograms in machine learning models.

	SHAP (Shapely Additive Explanations)	Dendrograms
Definition	SHAP values quantify the contribution of each feature to the prediction and provide local explanations for individual instances.	Dendrograms represent hierarchical clustering of model metrics, visualizing similarities and relationships among variables.
Interpretability	SHAP values offer interpretable feature importance scores, allowing understanding of the impact of each feature on the model’s predictions.	Dendrograms provide a visual representation of how variables cluster together based on their importance metrics, helping identify groups of similar features.
Feature Importance	SHAP values provide a quantitative measure of feature importance, indicating the extent to which a feature contributes to the prediction and how the model weighed the predictions positively or negatively for a certain covariate.	Dendrograms weight machine learning covariates based on model statistics such as gain, cover, and frequency, identifying which variables are most similar in terms of these metrics.
Individual Explanations	SHAP values provide explanations at the individual instance level, illustrating how each feature contributes to the prediction for a specific data point.	Dendrograms do not provide individual-level explanations but rather offer a holistic view of feature similarities and relationships in the dataset.
Transparency	SHAP values offer a transparent and understandable way to explain complex machine learning models, providing insights into the decision-making process.	Dendrograms enhance transparency by visualizing patterns and connections among features, aiding in model understanding.
Application	SHAP values are commonly used for feature selection, model debugging, and understanding the model’s behavior and predictions for specific instances.	Dendrograms are useful for identifying groups of similar features and exploring relationships between variables in a hierarchical manner based on model statistics.

The table highlights the complementary nature of dendrograms and SHAP values in understanding machine learning models. Dendrograms provide insights into the relationships and similarities among variables based on model metrics, emphasizing their collective behavior. In contrast, SHAP values focus on individual features, quantifying their importance and providing explanations at the instance level. Together, these approaches contribute to the interpretability and transparency of the models, enabling a comprehensive understanding of their predictions.

Where g(x’) represents the prediction of the explanation model for the specific coalition vector. *Φ*_0_ represents the intercept or bias term. *Φ*_*j*_ is the shaply value or feature attribution. $z_{j}^{\prime }$ represents the presence or absence of the feature in the model. The formulas deal primarily with each feature.

Eq 2 shows the general formula for calculating distances for dendrogram hierarchical clustering is as follows:

$$d_{mn}=\sqrt{\sum_{i=1}^{N}{\left(\mu_{mi}-\mu_{mi}\right)}^{2}}$$ (2)

Where *d*_*mn*_ represents the distances between features m and n. *i* is the layer number and *μ* is the mean of the feature being compared. N is the number of layers. Utilizing dendrograms allow for comparing the relationship between multiple features in their relationship with one another.

We utilize the MIMIC-III (Medical Information Mart for Intensive Care III) dataset, a large-scale electronic health record database, to deploy our machine learning model. By using SHAP, we can not only identify important covariates, but also visualize the direction in which the model is predicting their effects. By grouping important covariates by the commonly used metrics of Gain, Cover, and Frequency, we can assess the performance of each covariate in the model.

## Methods

The MIMIC-III database (V.1.4, 2016) contains de-identified data on 46,520 patients and 58,976 admissions to the ICU of the Beth Israel Deaconess Medical Center, Boston, USA, between June 1, 2001, and October 31, 2012. This publicly available critical care database provides comprehensive information on demographics, diagnoses, laboratory tests, medications, procedures, fluid balance, vital signs, radiology reports, and survival data. Approval to extract data from MIMIC-III after completing the National Institutes of Health Protecting Human Research Participants web-based training course was given via Certification Number: 28860101. The publicly available dataset was uploaded to Zenodo.

### Dataset and cohort selection

Our investigation utilized data from the MIMIC-III (V.1.4, 2016), which was created to provide researchers with a comprehensive and freely accessible dataset of critical care patients to advance clinical research, patient care, and medical education. The database contains de-identified information on tens of thousands of ICU patients, including demographics, diagnoses, laboratory results, medications, procedures, and more. This allows researchers to study and analyze clinical outcomes, treatment patterns, and other critical care-related topics using real-world data.

For this study, we included adult patients (≥15 years old) diagnosed with heart failure (HF) identified through manual review of ICD-9 codes. Two researchers conducted the code review, and they excluded patients without an ICU record or missing left ventricular ejection fraction (LVEF) or N-terminal pro-brain natriuretic peptide (NT-proBNP) data. Data was read into R programming and any individual without an outcome for heart failure was excluded (N = 1,176).

### Dependent variable

The study’s primary outcome was in-hospital mortality, which is defined as the survivors’ and non-survivors’ vital status at hospital discharge.

### Independent variable

For this study, data were extracted from the following tables in the MIMIC-III dataset: ADMISSIONS, PATIENTS, ICUSTAYS, D_ICD DIAGNOSIS, DIAGNOSIS_ICD, LABEVENTS, D_LABIEVENTS, CHARTEVENTS, D_ITEMS, NOTEEVENTS, and OUTPUTEVENTS. The variables were selected based on their clinical relevance, general availability at the time of presentation, and previous studies.

The extracted data included demographic characteristics such as age, sex, ethnicity, weight, and height, as well as vital signs such as heart rate, systolic blood pressure, diastolic blood pressure, mean blood pressure, respiratory rate, body temperature, saturation pulse oxygen, and urine output in the first 24 hours. Comorbidities including hypertension, atrial fibrillation, ischemic heart disease, diabetes mellitus, depression, hypoferric anemia, hyperlipidemia, chronic kidney disease (CKD), and chronic obstructive pulmonary disease (COPD) were also recorded. Laboratory variables such as hematocrit, red blood cells, mean corpuscular hemoglobin, mean corpuscular hemoglobin concentration, mean corpuscular volume, red blood cell distribution width, platelet count, white blood cells, neutrophils, basophils, lymphocytes, prothrombin time, international normalized ratio, NT-proBNP, creatine kinase, creatinine, blood urea nitrogen, glucose, potassium, sodium, calcium, chloride, magnesium, the anion gap, bicarbonate, lactate, hydrogen ion concentration, partial pressure of CO2 in arterial blood, and left ventricular ejection fraction (LVEF) were also extracted.

The calculated mean value of variable data with multiple measurements collected throughout the hospital stay was used in the analysis. Variables with missing data are common in the MIMIC-III, however, eliminating patients with incomplete data can bias the study. Therefore, imputation is an important step in data preprocessing. All screening variables contained <25% missing values. Multiple imputation was done to handle missing values.

### Model construction and statistical analysis

In univariate logistic models, the outcome of in-hospital mortality was used to identify covariates associated with each type. The machine learning model XGBoost was used because of its widespread use in the literature and improved predictive accuracy for healthcare predictions. Other studies using the NHANES cohort found that XGBoost offered the best balance between training efficiency, model accuracy, and transparency. The final set of model fit parameters (80:20) was calculated using a test and training set method. To determine the model’s fit, the area under the receiver operator characteristic curve (AUROC) was calculated.

### Model feature importance statistics and SHAP visualization

The frequency, gain, and coverage were calculated for model covariates to identify risk factors associated with in-hospital mortality and they were ranked according to their gain. The feature’s relative contribution to the model’s predictions is shown by the Gain metric, while the feature’s total number of observations is shown by the Cover metric. On the other hand, frequency indicates how frequently a feature appears in the machine-learning model’s trees. Gain was chosen as the primary metric for ranking covariates because it is easy to understand and simple. Gain is the proportion of a given covariate’s influence on the final prediction. The strongest connections between the risk of hospital mortality and continuous covariates were visualized using SHAP explanations.

### Dendrogram and heatmap creation based on gain, cover and frequency

Based on Gain, Cover, and Frequency, a dendrogram and heatmap were created. In the beginning, model covariates were ranked according to Gain, Cover, and Frequency in order to identify factors associated with hospital mortality. For each covariate, the Gain, Cover, and Frequency were calculated and sorted by value. The arranged covariate information was utilized to make a dendrogram that portrays the connection between different covariates in view of their comparability. Using Ward’s minimum variance method, the covariates were clustered based on their similarity to Gain, Cover, and Frequency for the dendrogram. The elbow method was used to figure out how many clusters were there, and k = 8 was chosen as the best number. The relationship between the covariates was then displayed on a heatmap alongside the hierarchical cluster. The goal of hierarchical cluster analysis is to create a tree diagram in which the items with the greatest degree of similarity are grouped together.

## Results

Table 2 shows the 1,176-heart failure and ICU patients that met the inclusion criteria in this study. Of those, 159 (13.5%) individuals had in hospital mortality and 1,017 (86.5%) did not. There was 558 (47.4%) males in the total cohort with 80 (50.3%) in the hospital mortality cohort and 478 (47%) in the no hospital mortality cohort. Average age in the cohort that was 74.05 (SD = 12.85) with the hospital mortality group having an average age of 76.24 (SD = 13.22) and the group without hospital mortality having an average age of 73.71 (SD = 13.46). Average urine output was 1899.28 (SD = 1272.36) mL/day with the hospital mortality group having 1345.97 (SD = 1136.58) mL/day and the group without hospital mortality having 1986.91 (SD = 1271.16) mL/day. The average leukocyte count in the cohort was 10.72 (SD = 5.23) cells per microliter. For the hospital mortality group the leukocyte count was 13.47 (SD = 7.42) cells per microliter and for the group without hospital mortality the leukocyte count was 10.28 (SD = 4.66) cells per microliter. The average bicarbonate value was 26.91 (SD = 5.17) mEq/L. Amongst the group with hospital mortality the average bicarbonate value was 24.00 (SD = 5.42) mEq/L. Amongst the group without hospital mortality the average bicarbonate value was 27.37 (SD = 4.98) mEq/L. The average platelet value was 241.52 platelets per microliter. For the group with hospital mortality the average platelet value was 216.21 platelets per microliter. For the group without hospital mortality the average platelet value was 245.47 platelets per microliter.

**Table 2:** Demographic variables.

	Total	Hospital Mortality	No Hospital Mortality	p-values
Total	1176	159 (0.135)	1017(0.865)	
age	74.05 (13.44)	76.24 (13.22)	73.71 (13.45)	0.026
male	558 (0.474)	80 (0.503)	478 (0.470)	0.439
BMI	30.19 (9.33)	28.61 (9.92)	30.40 (9.23)	0.07
hypertensive	844 (0.718)	101 (0.635)	743 (0.731)	0.02
atrialfibrillation	531 (0.452)	92 (0.579)	439 (0.432)	0.001
CHD with no MI	101 (0.086)	12 (0.075)	89 (0.088)	0.598
diabetes	495 (0.421)	57 (0.358)	438 (0.431)	0.081
deficiencyanemias	399 (0.339)	35 (0.220)	364 (0.358)	p<0.001
depression	140 (0.119)	11 (0.069)	129 (0.127)	0.012
Hyperlipemia	447 (0.380)	50 (0.314)	397 (0.390)	0.059
Renal failure	429 (0.365)	37 (0.233)	392 (0.385)	p<0.001
COPD	89 (0.076)	7 (0.044)	82 (0.081)	0.048
heart rate	84.58 (16.02)	89.82 (15.25)	83.75 (15.99)	p<0.001
Systolic blood pressure	118.00 (17.37)	112.18 (16.61)	118.91 (17.32)	p<0.001
Diastolic blood pressure	59.53 (10.68)	57.18 (8.88)	59.90 (10.90)	0.001
Respiratory rate	20.80 (4.00)	21.98 (4.42)	20.62 (3.90)	p<0.001
temperature	36.68 (0.61)	36.54 (0.70)	36.70 (0.59)	0.006
SP O2	96.27 (2.30)	95.86 (3.10)	96.34 (2.14)	0.064
Urine output	1899.28 (1272.36)	1345.97 (1136.58)	1986.91 (1271.16)	p<0.001
hematocrit	31.91 (5.20)	31.69 (5.27)	31.94 (5.19)	0.576
RBC	3.57 (0.63)	3.54 (0.70)	3.58 (0.62)	0.455
MCH	29.54 (2.62)	29.63 (2.87)	29.53 (2.58)	0.672
MCHC	32.86 (1.40)	32.75 (1.36)	32.88 (1.41)	0.265
MCV	89.90 (6.54)	90.47 (7.16)	89.81 (6.43)	0.28
RDW	15.95 (2.13)	16.75 (2.33)	15.83 (2.07)	p<0.001
Leucocyte	10.72 (5.23)	13.47 (7.42)	10.28 (4.66)	p<0.001
Platelets	241.52 (113.17)	216.21 (129.64)	245.47 (109.92)	0.008
Neutrophils	80.12 (11.14)	82.12 (14.77)	79.79 (10.41)	0.072
Basophils	0.41 (0.47)	0.37 (0.67)	0.41 (0.43)	0.564
Lymphocyte	12.23 (8.64)	9.24 (9.82)	12.71 (8.34)	p<0.001
PT	17.49 (7.39)	20.09 (11.18)	17.07 (6.50)	0.001
INR	1.63 (0.83)	1.93 (1.27)	1.58 (0.73)	0.001
NT-proBNP	11011.04 (13153.83)	15037.14 (16461.49)	10381.59 (12450.97)	0.001
Creatine kinase	246.94 (1485.25)	535.59 (3688.87)	202.08 (657.54)	0.295
Creatinine	1.64 (1.28)	1.79 (1.12)	1.62 (1.30)	0.075
Urea nitrogen	36.29 (21.86)	47.51 (27.96)	34.54 (20.21)	p<0.001
glucose	148.80 (51.49)	153.01 (57.63)	148.14 (50.46)	0.317
Blood potassium	4.18 (0.41)	4.32 (0.53)	4.15 (0.39)	p<0.001
Blood sodium	138.90 (4.15)	138.20 (5.02)	139.01 (3.98)	0.055
Blood calcium	8.50 (0.57)	8.24 (0.62)	8.54 (0.55)	p<0.001
Chloride	102.29 (5.33)	103.10 (6.00)	102.17 (5.21)	0.065
Anion gap	13.92 (2.65)	15.46 (3.52)	13.68 (2.41)	p<0.001
Magnesium ion	2.12 (0.25)	2.17 (0.29)	2.11 (0.24)	0.025
PH	7.38 (0.07)	7.36 (0.07)	7.38 (0.06)	p<0.001
Bicarbonate	26.91 (5.17)	24.00 (5.42)	27.37 (4.98)	p<0.001
Lactic acid	1.85 (0.98)	2.39 (1.43)	1.76 (0.85)	p<0.001
PCO2	45.54 (12.71)	44.11 (12.67)	45.81 (12.71)	0.142
EF	48.71 (12.87)	47.92 (14.77)	48.83 (12.55)	0.463

Descriptive statistics for demographic characteristics and all covariates within the machine learning model, stratified by hospital mortality.

**Fig 1:**
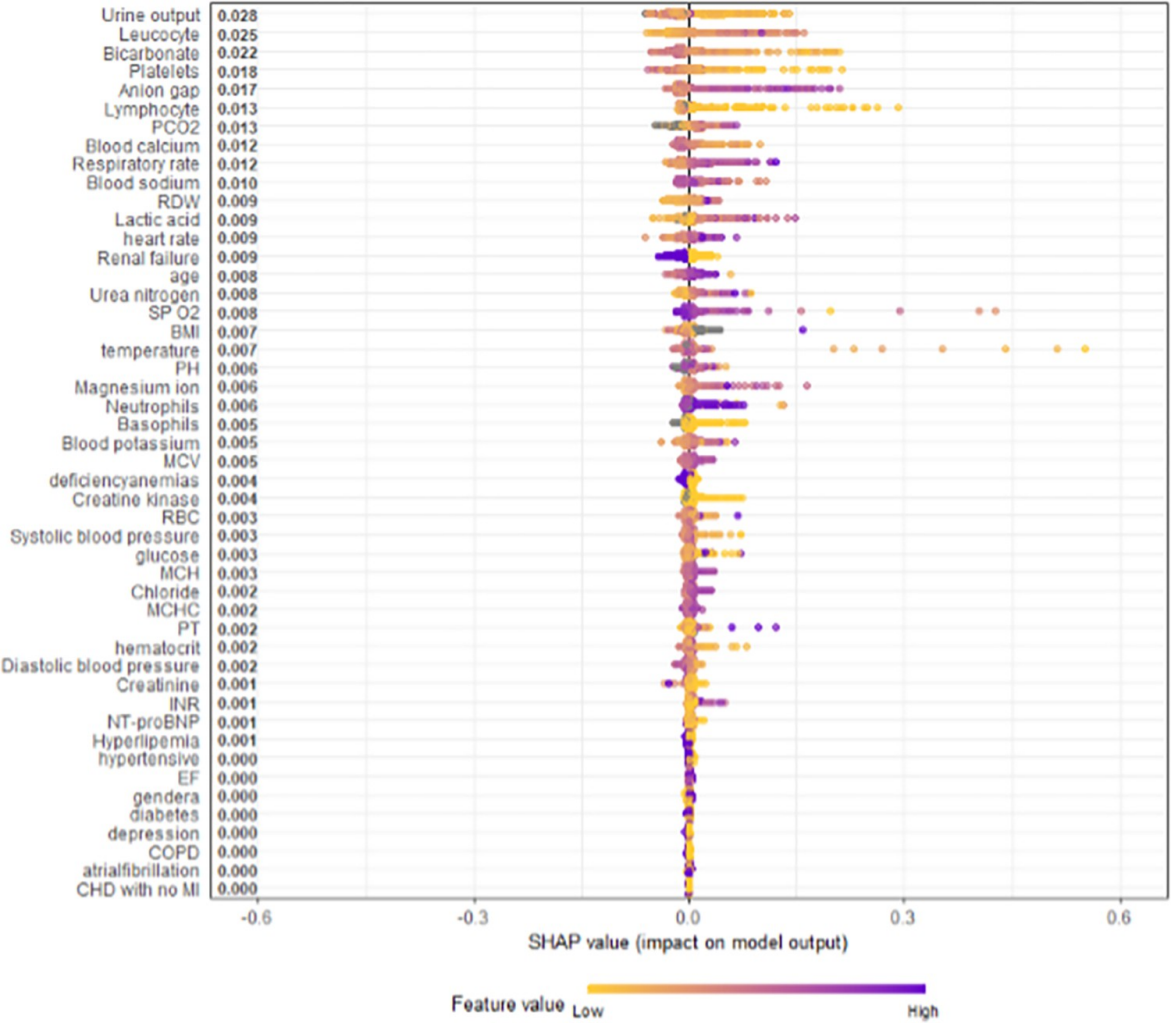
Overall SHAP explanations. SHAP explanations, purple color representing higher values of the covariate while yellow representing lower values of the covariate. X-axis is the change in log-odds for inpatient hospital mortality.

Fig 1 displays the overall SHAP explanations for all the model covariates fit to predict in-hospital mortality to patients in heart failure patients in the intensive care unit. The model had a AUROC = 0.662.

**Fig 2:**
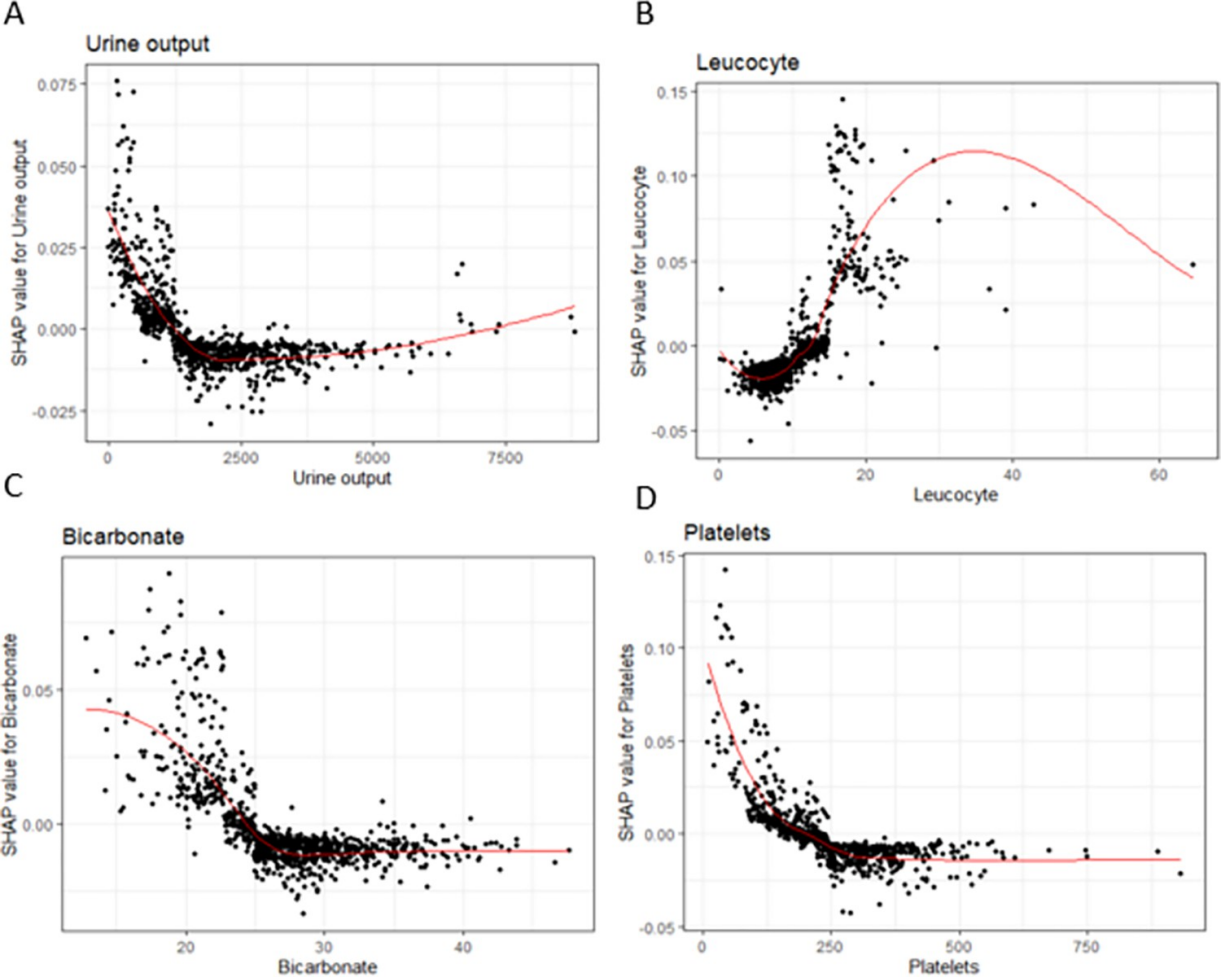
SHAP explanations for the Top 4 continuous covariates sorted by overall SHAP explanations. SHAP explanations, covariate value on the x-axis, change in log-odds on the y-axis, red line represents the relationship between the covariate and log-odds for Hospital Mortality, each black dot represents an observation. Covariates: top left–Urine Output, top right–Leukocyte Count bottom left–Bicarbonate, bottom right–Platelets.

Fig 2 depicts the SHAP visualizations for the four most significant continuous covariates based on overall SHAP explanations. Our findings indicate that increasing urine output up to 1,250 mL/day was associated with decreased inpatient hospital mortality in heart failure patients in the intensive care unit. Additionally, increased leukocyte count up to 20 cells per microliter was associated with increased inpatient hospital mortality in heart failure patients in the intensive care unit. Increased bicarbonate count up to 25 mEq/L was associated with decreased inpatient hospital mortality in heart failure patients in the intensive care unit. Increasing platelet count up to 200 platelets per microliter was associated with decreased inpatient hospital mortality in heart failure patients in the intensive care unit.

Furthermore, Table 3 highlights the four top-ranked features by gain, which is a measure of the percentage contribution of the covariate to the overall model prediction. The most significant features were Bicarbonate (Gain = 6.7%), Platelets (Gain = 5.2%), Urine output (Gain = 5.1%), and temperature (Gain = 5%).

**Table 3:** Model gain statistics.

Feature	Gain	Cover	Frequency
Bicarbonate	0.067	0.039	0.027
Platelets	0.052	0.049	0.042
Urine output	0.051	0.046	0.035
temperature	0.050	0.078	0.050
Anion gap	0.050	0.039	0.027
Leucocyte	0.046	0.061	0.034
Lactic acid	0.045	0.025	0.035
SP O2	0.042	0.061	0.040
Lymphocyte	0.042	0.053	0.031
PCO2	0.034	0.027	0.030
Respiratory rate	0.033	0.033	0.029
BMI	0.032	0.032	0.058
heart rate	0.027	0.019	0.036
age	0.025	0.028	0.060
Neutrophils	0.024	0.031	0.027
Blood sodium	0.024	0.043	0.031
RDW	0.022	0.020	0.022
Blood potassium	0.022	0.018	0.022
PH	0.021	0.021	0.022
Urea nitrogen	0.021	0.032	0.023
Renal failure	0.021	0.010	0.014
Blood calcium	0.020	0.036	0.022
RBC	0.019	0.013	0.018
Systolic blood pressure	0.019	0.017	0.026
Creatine kinase	0.019	0.014	0.020
Magnesium ion	0.019	0.027	0.021
Basophils	0.018	0.010	0.017
PT	0.016	0.012	0.015
hematocrit	0.015	0.012	0.020
MCV	0.015	0.011	0.016
glucose	0.013	0.023	0.021
Creatinine	0.012	0.004	0.012
Diastolic blood pressure	0.011	0.004	0.020
Chloride	0.009	0.010	0.012
MCH	0.009	0.007	0.011
NT-proBNP	0.008	0.003	0.009
MCHC	0.007	0.004	0.009
INR	0.006	0.009	0.008
deficiencyanemias	0.003	0.013	0.006
EF	0.002	0.001	0.003
Hyperlipemia	0.002	0.000	0.003
hypertensive	0.002	0.001	0.005
gendera	0.001	<0.001	0.004
diabetes	0.001	<0.001	0.003
depression	0.001	<0.001	0.001
atrialfibrillation	0.001	<0.001	0.002
COPD	0.000	<0.001	0.001
CHD with no MI	0.000	<0.001	0.001

The Gain, Cover, and Frequency of all covariates within the XGBoost model. The Gain represents the relative contribution of the feature to the model and is the most important metric of model importance within this study. Covariates ordered according to the Gain statistic. AUROC was found to be 0.662.

**Fig 3:**
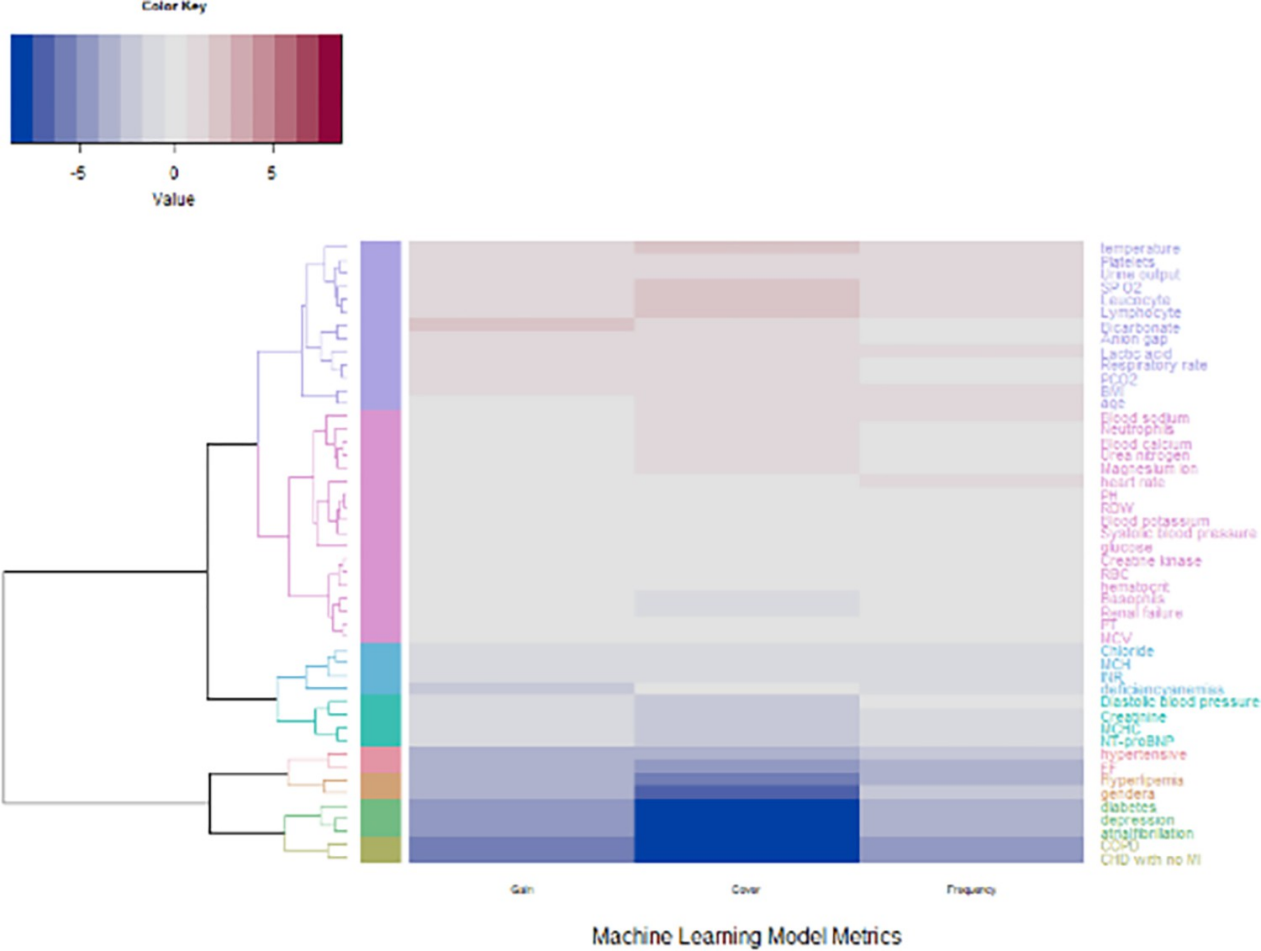
Dendrogram and heatmap of covariates grouped by cover, frequency, and gain. The colors of the dendrogram represent different groups and clusters of covariates based on their similarity in terms of the machine learning model metrics of cover, frequency, and gain. The length of the branches represents the degree of similarity between the covariates. Longer branches indicated greater differences between the covariates, and the closer together the branches are, the more similar covariates are. Colors of the heatmap show values of gain, cover, and frequency with red representing larger values and blue representing smaller values.

Fig 3 shows that in cluster 1 of the heatmap and dendrogram Temperature, platelets, urine output, Saturation of partial pressure of Oxygen (SPO2), Leukocyte count, lymphocyte count, bicarbonate, anion gap, respiratory rate, PCO2, BMI, and age were most similar in having high aggregate gain, cover, and frequency metrics.

## Discussion

In this retrospective, cross sectional cohort of heart failure patients in the ICU, a machine learning model to mortality had a AUROC of 0.662. 1,176 patients with heart failure and ICU admission who met the study’s inclusion criteria. Out of these patients, 159 (13.5%) had in-hospital mortality, while 1,017 (86.5%) did not. Of the total cohort, 558 (47.4%) were males, with 80 (50.3%) in the hospital mortality group and 478 (47%) in the no hospital mortality group. The average age of the cohort was 74.05 (SD = 12.85), with the hospital mortality group having an average age of 76.24 (SD = 13.22) and the group without hospital mortality having an average age of 73.71 (SD = 13.46).

The model achieved an area under the receiver operating characteristic curve (AUROC) of 0.662, which suggests a moderate predictive accuracy. The study included 1,176 heart failure patients admitted to the ICU, out of which 13.5% experienced in-hospital mortality. The results indicate that age, urine output, leukocyte count, bicarbonate value, and platelet count were different between patients who experienced in-hospital mortality and those who did not and are consistent with other studies [21,27–30]. Likewise, the findings that the hospital mortality group had a higher average age, lower urine output, higher leukocyte count, lower bicarbonate value, and lower platelet count compared to the group without hospital mortality are consistent with other studies [31–34].

Machine learning models have been successful in heart failure and at detecting mortality. Heart failure mortality has been looked at in patients requiring different levels of care from step-down care, progressive care, and intensive care to different stages and with many different covariates [35–38]. Researchers have utilized methods from logistic regression to machine learning [39–44]. Within machine learning researchers are starting to utilize transparent methods for visualization [24,45].

A popular method that increases understandability of machine learning models is SHAP [22,46]. We use transparent machine learning methods to detect real signals that are in line with our current understandings as described in literature and clinical practice. The SHAP visualizations further support the increased predictive power of these non-parametric methods by demonstrating their ability to accurately capture the non-linear interactions between covariates, without overfitting the model to achieve greater accuracy.

We further introduce another way to visualize machine learning statistics. Dendrograms and heat maps are commonly used in various fields such as biology, ecology, genetics, and data science to visualize relationships and clusters [47]. Heat maps are particularly useful in condensing large amounts of information into a concise visual representation, and have been applied to gene expression, sequencing, geographic data, and population densities [48]. To better understand the complex relationships described by machine learning models, we proposed utilizing dendrograms and heat maps to describe the gain, cover, and frequency of covariates [49,50]. We found that the cluster produced by the dendrogram is similar to that produced by the SHAP value. Dendrograms provide additional insights in the context of explainable machine learning by visually displaying the relationship between variables based on their similarity, which can help identify important variables and uncover patterns in the data. In hierarchical clustering, variables are grouped together based on their similarity, with variables that are more similar being grouped together in the same cluster. Dendrograms can help identify clusters of variables that are highly correlated with each other, which can be useful in identifying variables that may be driving the model’s predictions or outcomes. Additionally, dendrograms can help identify variables that are not strongly correlated with any other variables, which can suggest that these variables may not be very informative for the model. Overall, dendrograms can provide a useful visual aid for interpreting the relationships between variables in an explainable machine learning context. The choice of using gain, cover, and frequency to cluster the variables was based on their importance in the XGBoost algorithm. These metrics provide valuable information about the predictive power of each variable and their contribution to the model’s overall performance. While two features with disparate tree positions may share similar gain scores, the clustering is based on overall similarity across all three metrics. Additionally, the dendrogram provides a visual representation of how the variables are clustered, which can aid in interpretation and further analysis. We acknowledge that there may be alternative approaches to clustering and welcome further discussion on this topic.

One direction of research with dendrograms is focused on exploring associations between outcomes and multiple variables, rather than just examining the relationship with a single variable. Dendrograms can be instrumental in identifying patterns and relationships among various factors, aiding in the creation of ordered sets or guidelines in fields such as medicine. For instance, in the context of heart failure, a dendrogram analysis can incorporate multiple features like RBC count, Troponin, BNP, Cr, BUN, and an echocardiogram, revealing the interconnections and similarities between these variables to inform the development of comprehensive and structured order sets for managing heart failure patients.

Depending on the specific problem, different statistics such as gain, cover, or frequency may hold more significance. Gain assesses the relative impact of each covariate on the model’s accuracy, which is especially relevant when high accuracy is paramount. Frequency measures the frequency with which a covariate appears in the model’s decision trees, providing insight into the trees’ patterns. Cover measures the number of instances of a given covariate, promoting generalizability. Since all three statistics hold importance and their relevance may vary depending on the problem, it is essential to develop a workflow that allows the modeler to evaluate how the machine learning algorithm weighs each metric. This facilitates feature selection.

### Limitations

This machine-learning analysis has a retrospective nature, which may introduce bias. However, the potential bias was minimized by using training and testing sets to avoid overfitting. It is important to acknowledge this limitation. The use of SHAP visualizations can assist researchers in distinguishing whether the effects of each covariate are the result of true signal or noise, thereby reducing the risk of type-1 errors. Despite these limitations, we believe that machine-learning can serve as a useful preliminary measure in identifying potential risk factors. Subsequently, clinicians can evaluate these factors further based on the patient’s individual clinical presentation.

### Conclusion

Machine learning models can find significant predictors for inpatient mortality in critically hospitalized heart failure patients. Feature importance with SHAP generates associations consistent with literature. Dendrograms and heat maps provide useful tools for model understandability.

## Abbreviations

MIMIC-III: Medical Information Mart for Intensive Care
SHAP: Shapely Additive Explanations
AUROC: Area under Receiver Operating Characteristic Curve
HF: Heart Failure
ICD-9: International Classification of Diseases
ICU: Intensive Care Unit
MIMIC-III: Medical Information Mart for Intensive Care
SHAP: Shapely Additive Explanations
